# The interplay between diabetes and non-alcoholic fatty liver disease: a cross-sectional study from Pakistan

**DOI:** 10.1097/MS9.0000000000001875

**Published:** 2024-02-28

**Authors:** Ayesha Akhtar, Huda Ijaz, Maria Waseem, Muhammad Ijaz Khan, Yasir Saif, Haris Iqbal, Syeda Aasia Batool, Usha Kumari, Salim Surani, Aarash Khan

**Affiliations:** a Internal Medicine, Khyber Teaching Hospital, Peshawar; b Internal Medicine, Allama Iqbal Medical College; c King Edward Medical University, Lahore; d Dow University of Health Sciences; e Medicine, Dow University of Health Sciences, Karachi; f Nishtar Medical university, Multan, Pakistan; g Medicine Unit, Naas General Hospital, Kildare; h Internal Medicine, University Hospital Kerry, Kerry, Ireland; i Texas A&M University, College Station, TX; j Research Collaborator, Mayo Clinic, Rochester, MN; k Herat University, Herat, Afghanistan

**Keywords:** alanine, glycated haemoglobin, NASH, non-alcoholic, noninsulin dependent diabetes mellitus, steatohepatitis, transaminase

## Abstract

**Background and objectives:**

Non-alcoholic fatty liver disease (NAFLD) is characterized by ectopic deposition of fat in the liver, in the absence of other secondary causes of fat buildup. The relationship between NAFLD, including alanine aminotransferase (ALT), and glycated haemoglobin (HbA1c), is important for predicting the severity of disease and prognosis. This study aims to investigate the association of HbA1c in type 2 diabetes mellitus (T2DM) patients with NAFLD via measuring the ALT levels.

**Materials and methods:**

This retrospective cross-sectional study enroled 130 patients with T2DM and NAFLD. The association between levels of HbA1c and ALT in patients of NAFLD with controlled and uncontrolled T2DM, respectively, was investigated. Stratification was done based on gender and diabetic control, using HbA1c levels as a marker of glycemic control. Serum ALT levels were also compared in both groups.

**Results:**

The mean age of the participants was 50.2±5.7 years. The total participants were 130, of which 77 (59.3%) were females and 53 (40.7%) were males. The numbers of patients having uncontrolled T2DM (HbA1c>7%), and controlled T2DM (HbA1c <7%) were 78 (60%) and 52 (40%), respectively. Moreover, 46 (35.3%) females and 32 (24.7%) males had uncontrolled T2DM, and 31 (23.8%) females and 21 (16.2%) males had controlled T2DM. The mean ALT level for uncontrolled and controlled T2DM in female patients was found to be 24.6±3.4 and 13.5±2.4, respectively, (*P* <0.05). For male patients, it was found to be 54.0±4.9 and 29.1±5.4, respectively (*P*=0.008).

**Conclusion:**

There is a positive association between elevated HbA1c and ALT levels in T2DM patients with NAFLD.

## Introduction

Highlights

- The study aims at bringing forward an association between type 2 diabetes mellitus (T2DM) and non-alcoholic fatty liver disease (NAFLD) in patients of T2DM with deranged alanine aminotransferase (ALT).
- The study puts forward a positive association between elevated glycated haemoglobin (HbA1c) levels and ALT levels in patients of T2DM with NAFLD.
- Literature suggests that HbA1c levels can contribute to progression of NAFLD and deranged liver function enzymes can also contribute to the onset of T2DM indicating a bidirectional association.
- The bidirectional association between T2DM and NAFLD puts forward a possibility of early diagnosis and timely management of one condition if early screening is employed in a diagnosed patient of the other condition, minimizing morbidity.

Non-alcoholic fatty liver disease (NAFLD), a growing public health concern, ranges from NAFLD to non-alcoholic steatohepatitis (NASH). Minor fatty infiltration can occur in the hepatocytes of a healthy individual, but it becomes pathological when at least 5% of hepatocytes are affected^1^. The absence of alcohol exposure is the only factor that distinguishes it from alcoholic liver disease (ALD). NAFLD is a prevalent condition, affecting approximately one-quarter of the global population^2^. Management plans for NAFLD are majorly constructed according to disease severity and the presence of co-morbidities. Studies show that NAFLD can progress to well-defined NASH with bridging fibrosis^3^. A cohort study found that the disease usually progresses gradually and progressively, especially in patients with diabetes, until advanced fibrosis develops in the patients^4^. Compared to the general population, which has a prevalence of 5%, patients with type 2 diabetes mellitus (T2DM) have a much greater rate of NAFLD, ranging from 25 to 75%^5^. A meta-analysis of 80 studies involving 49419 T2DM individuals revealed a prevalence of NAFLD of 55.5%^6^. Another study conducted on outpatient T2DM patients with steatosis screening shows a 70% prevalence of NAFLD (including early steatosis)^7^. NAFLD and T2DM have a bidirectional connection because they both share the common pathogenesis of insulin resistance^5,6^. The markers of liver function and indicators of the degree of liver injury, aspartate transaminase (AST), and alanine aminotransferase (ALT) levels have a positive association with T2DM and NAFLD^6^. According to research, the high prevalence of NAFLD and high glycated haemoglobin (HbA1c) levels are directly correlated^8^. Despite these studies, a gap exists in assessing the extent of liver damage in patients having both NAFLD and T2DM regarding their glycemic control. The common markers for liver damage are ALT and AST which are found in high concentrations inside the liver. Aspartate Aminotransferase is also present in red blood cells, the brain, the kidney, skeletal muscle, and the heart, and ALT is present in low concentrations inside the kidney and skeletal muscle^9^. Thus, raised levels of ALT are more specific for liver damage^10^. Therefore, this study focuses on the association of ALT levels as they are more indicative of the extent of liver damage. Thus, this study is unique in establishing a relationship between the levels of ALT and HbA1c to assess the extent of liver damage depending upon the glycemic control.

## Methods

An analytical retrospective, cross-sectional study was carried out from November 2022 to February 2023, in a tertiary care hospital. All patients included in the study were diagnosed patients of T2DM. All the patients included presented in Internal Medicine and Endocrinology outpatient departments. The study was designed to evaluate the association of ALT and HbA1c levels in patients of NAFLD with controlled and uncontrolled T2DM, respectively.

Researchers initially employed 130 patients with ultrasound-detected fatty liver together with either controlled (HbA1c<7%) or uncontrolled (HbA1c>7%) T2DM. Sampling technique utilized was non-randomized systemic smapling where first 100 patients with T2DM and deranged ALT were considered. The source of data collected is HMIS system of Khyber Teaching Hospital. All patients with a history of chronic liver disease, alcohol intake, viral hepatitis, hemochromatosis, and Wilson’s disease were excluded. The presence or absence of dyslipidemia or obesity was not taken into account. ALT and HbA1c levels were compared in patients with controlled and uncontrolled T2DM, respectively.

NAFLD was identified using abdominal ultrasound (US) despite liver biopsy being the gold standard for the diagnosis because of its recent replacement by other screening techniques worldwide^11^. US scans were funded by the government as the study was carried in a public sector healthcare setting. HbA1c levels were measured by immunoassay technique.

Analysis was conducted using IBM SPSS Statistics version 25.0. Mean and standard deviation (SD) were used to describe continuous variables, such as age and ALT level, whereas frequency and percentages were used for categorical variables, such as gender and uncontrolled and controlled diabetes stratification. The χ^2^ test was conducted to assess the statistical significance of the relationship between the variables, and a *P* value of less than 0.05 was considered statistically significant. Participation in the study was voluntary and informed consent was obtained before data collection from all the participants enroled in the study.

## Results

A total of 130 participants having T2DM with NAFLD participated in the study. The mean age of the participants was found to be 50.2±5.7 years. Females comprised 59.7% and males comprised of 40.3% of the population. The study revealed that 60% participants (*n*=78) had uncontrolled T2DM while 40% participants (*n*=52) had controlled T2DM.

Stratification of ALT levels was carried out with gender distribution in uncontrolled and controlled T2DM groups, as shown in Table 1, with a statistically significant *P* value of 0.008 for male patients and *P* value less than 0.05 for female patients.

**Table 1:** Stratification of alanine aminotransferase level to gender (*n*=130)

Sex^a^	HbA1c parameter	Frequency	Mean±SD ALT levels
Female	HbA1c<7	31	13.52±2.42
	HbA1c>7	46	24.6±3.47
Male	HbA1c<7	21	29.19±5.45
	HbA1c>7	32	54.03±4.92

ALT, alanine aminotransferase; HbA1c, glycated haemoglobin.

a *P* value<0.008.

## Discussion

More than 451 million individuals are affected by T2DM globally^5^ while Pais *et al.* ^3^ suggest that a significant proportion of patients with NAFLD progress to hepatic fibrosis on deterioration of metabolic risk factors such as diabetes. T2DM and NAFLD commonly exist together. It has been regarded as a manifestation of the metabolic syndrome. A study done on outpatient patients with T2DM undergoing screening for steatosis demonstrated a 70% prevalence of NAFLD (including early steatosis)^7^. From an aetiological perspective, in addition to genetic predisposition, obesity, and T2DM have been found to contribute considerably to the increased incidence of NAFLD in diabetic and pre-diabetic patients in the last two decades^12^. Varying degrees of derangement in ALT among diabetic patients have been reported, with the prevalence of NAFLD being increased from 27 to 73%, correlating with the increase in HbA1c levels from 6 to 8.5% concurrently. Along with these findings, higher triglyceride levels were also found to be associated with higher HbA1c values^8^. Our investigation revealed comparable results, indicating that patients with HbA1c levels exceeding 7% exhibited increased transaminase activity.

NAFLD is a significant global health concern, a multifactorial disease influenced by several genetic and environmental factors. In addition to the association of T2DM to NAFLD and NASH, evidence supports that raised HbA1c in T2DM indicates abnormal glucose tolerance, which may be one of the key factors contributing to the hepatic alterations manifesting in biopsy-proven NAFLD together with increased ALT in patients of metabolic syndrome (T2DM, central obesity, dyslipidemia, and hypertension)^13^. Another clinical study indicates that raised HbA1c can also contribute to NAFLD progression either directly by stimulating a receptor protein RAGE (receptor for advanced glycation end-products) or indirectly by promoting hypoxia and suppressing nitric oxide release^14^.

On the other hand, a cohort study on 5000 Chinese individuals revealed that elevated liver function enzymes are associated with the onset of T2DM, irrespective of the presence of any conventional risk factors^15^ bringing forward a finding that complies with our study. The close relationship found between the two may be explained by the involvement of activation of certain transcription factors, hepatic fat accumulation, alterations in energy metabolism, and several inflammatory signalling pathways observed in the parallel chronic and subclinical nature of disease progressions and subsequent development of insulin resistance and obesity^16^. Extrapolation of the clinical implication of the link found between obesity, T2DM, and NAFLD leads to the development of the HAALT scoring system in a study^17^ to predict the progression of NAFLD into NASH in obese patients. The system utilized five significant independent factors obtained from binary logistic regression analysis, namely HbA1c, AST, ALT, liver span in the US, and serum triglycerides. The study found that the HAALT system is a useful tool in identifying patients who require intensive therapy. This investigation supports our study’s findings that ALT and HbA1c are related in NAFLD patients. Together with this, Masaaki Sagara et. Al suggests a strong relationship between increased serum dipeptidyl peptidase-4 (sDPP-4) in T2DM and the severity of hepatic fibrosis evaluated by liver span measurement (LSM) and Fibroscan-AST score (FAST score) measured by transient elastography^18^ indicating another possible explanation for the findings of our study.

Recent evidence indicates a compelling association between elevated transaminase levels and difficulty in achieving glycemic targets in diabetics. The persistence of high liver enzyme levels may predict declining beta-cell function, which may be attributed to insulin resistance^19^ and may help in the timely consideration of the need for a change in the therapeutic regimen. High insulin resistance or low insulin secretion boosts lipase activity, on the other hand, causing excess free fatty acids that the liver cannot oxidize, leading to NAFLD^11^. Thus, signs of ketogenesis in patients of uncontrolled T2DM, may indicate the importance of inculcating monitoring of liver function in routine laboratory investigations for timely diagnosis of developing NAFLD. A five-fold higher prevalence of NAFLD is reported in patients of T2DM as compared to non-diabetic patients^16^. The complicated and bidirectional connection between T2DM and NAFLD results in a harmful cycle of progressive damage. This highlights the importance of early screening for liver disease in individuals with T2DM, and physicians should consider a lower threshold for screening patients with T2DM for NAFLD/NASH^7^.

Together with the need for more efficient screening, several therapeutic implications can be drawn from the clinical association between T2DM and NAFLD supported by our study. Several studies suggest that therapeutic strategies aimed at reducing visceral fat and increasing adiponectin levels can provide remarkable benefits in improving hepatic function in patients with T2DM. A study has found remarkable potential for hepatic steatosis improvement and attenuation of liver fibrosis in patients of T2DM and NAFLD with the administration of dapagliflozin, a sodium-glucose co-transporter-2 inhibitor, a drug normally prescribed for the treatment of T2DM^20^. In addition, liver function improvement with dapagliflozin is also found to be associated with a concomitant decrease in sDDP-4 levels by decreasing visceral fat, suggesting sodium-glucose co-transporter-2 as an effective therapeutic strategy for patients suffering from T2DM and NAFLD concurrently^21^. Another oral hypoglycaemia drug named, pioglitazone, a thiazolidinedione, is found to exhibit equally beneficial effects in improving liver function in similar patients by increasing adiponectin levels^22^. In addition to these, metformin, a biguanide, often prescribed as a first-line oral hypoglycaemia drug for the treatment of T2DM, is found to have remarkable therapeutic implications for the treatment of steatohepatitis and hepatic fibrosis by attenuation of hepatic stellate cell activation^23^. Together with this, according to a 6-year follow-up study on a new medication administered to newly diagnosed T2DM patients, those receiving triple treatment (metformin, exenatide, and pioglitazone) had less hepatic fibrosis and steatosis than those receiving stepwise conventional therapy (metformin → glipizide → glargine insulin)^24^. We can improve the general health outcomes of people with diabetes and related complications by identifying and treating the disease as soon as possible, and the findings of our study establish both the need and proof to reconsider the threshold to screen patients for NAFLD who do not have adequate glycemic control, and to pay more attention to ALT as its derangement indicates the possible future onset/progression of NASH.

### Limitations

Firstly, a cross-sectional study is a snapshot in time, and therefore it cannot establish temporal relationships between variables; diabetes and NAFLD. Secondly, this study cannot establish causality as we only measured associations between variables. The presence or absence of dyslipidemia or obesity, lifestyle, dietary habit and other comorbid conditions were not taken into account, which limits its generalizability. Therefore, further studies, such as longitudinal studies or randomized controlled trials, may be required to establish causality. Moreover, this study is limited to a small sample size from the Pakistani population and may not be generalizable to other populations or regions and the small size of the sample reders them underpowered. Finally, the presence or absence of other co-morbidities like dyslipidemia and/or obesity and use of drugs which cause liver injury can affect the results of the study too. Thus, more studies incorporating other variables, co-morbidities, and more population should be conducted.

## Conclusion

In conclusion, the findings of this study highlight a positive relationship between HbA1c and ALT in diabetic patients with NAFLD. Patients with T2DM should have their transaminase levels routinely monitored and, clinicians should be on the lookout for the presence of NAFLD to provide the most effective and appropriate treatment.

## STROCCS criteria

This manuscript has been reported in line with the STROCCS criteria^25^.

## Ethical statement

Ethical approval was obtained from the ethical board of the corresponding institute before starting this article.

## Consent

Written informed consent was obtained from the patient for publication and any accompanying images. A copy of the written consent is available for review by the Editor-in-Chief of this journal on request.

## Source of funding

Not applicable.

## Author contribution

Idea and conceptualization: A.A. Data collection and analysis: S.B., S.S. Writing—original draft: H.I., M.W. Writing—review and editing: M.K., Y.S. Proofreading: H.I. Project administration: A.K., U.K.

## Conflicts of interest disclosure

The authors report no actual or potential conflicts of interest or financial disclosure.

## Research registration unique identifying number (UIN)

Researchregistry9270.

## Guarantor

Ayesha Akhtar.

## Data statement

Data associated with this submission can be acquired upon request from the corresponding author.

## Provenance and peer review

Not commissioned, externally peer-reviewed.

## References

[1] Sharma B John S. Nonalcoholic Steatohepatitis (NASH). 2023 Apr 7. In: StatPearls [Internet]. StatPearls Publishing; 2023. 29262166

[2] Lee CH Lui DT Lam KS. Non-alcoholic fatty liver disease and type 2 diabetes: an update. J Diabetes Investig 2022;13:930–940. 10.1111/jdi.13756 PMC9153839 35080136

[3] Pais R Charlotte F Fedchuk L. LIDO Study Group. A systematic review of follow-up biopsies reveals disease progression in patients with non-alcoholic fatty liver. J Hepatol 2013;59:550–556. 23665288 10.1016/j.jhep.2013.04.027

[4] McPherson S Hardy T Henderson E. Evidence of NAFLD progression from steatosis to fibrosing-steatohepatitis using paired biopsies: implications for prognosis and clinical management. J Hepatol 2015;62:1148–1155. 25477264 10.1016/j.jhep.2014.11.034

[5] Chen L Jiang L. Clinicopathologicalfeatures and related risk factors of Type-2 diabetes mellitus complicated with nonalcoholic fatty liver. Pak J Med Sci 2022;38:1771–1775. 36246713 10.12669/pjms.38.7.6289 PMC9532671

[6] Younossi ZM Golabi P de Avila L. The global epidemiology of NAFLD and NASH in patients with type 2 diabetes: a systematic review and meta-analysis. J Hepatol 2019;71:793–801. 31279902 10.1016/j.jhep.2019.06.021

[7] Lomonaco R Godinez Leiva E Bril F. Advanced liver fibrosis is common in patients with type 2 diabetes followed in the outpatient setting: the need for systematic screening. Diabetes Care 2021;44:399–406. 33355256 10.2337/dc20-1997 PMC7818321

[8] Portillo-Sanchez P Bril F Maximos M. High prevalence of nonalcoholic fatty liver disease in patients with type 2 diabetes mellitus and normal plasma aminotransferase levels. J Clin Endocrinol Metab 2015;100:2231–2238. 25885947 10.1210/jc.2015-1966 PMC6287506

[9] Wroblewski F. The clinical significance of alterations in transaminase activities of serum and other body fluids. Adv Clin Chem 1958;1:313–351. 13571034

[10] Giannini EG Testa R Savarino V. Liver enzyme alteration: a guide for clinicians. CMAJ 2005;172:367–379. 15684121 10.1503/cmaj.1040752 PMC545762

[11] Afolabi BI Ibitoye BO Ikem RT. The relationship between glycaemic control and non-alcoholic fatty liver disease in nigerian type 2 diabetic patients. J Natl Med Assoc 2018;110:256–264. 29778128 10.1016/j.jnma.2017.06.001

[12] Stefan N Cusi K. A global view of the interplay between non-alcoholic fatty liver disease and diabetes. Lancet Diabetes Endocrinol 2022;10:284–296. 35183303 10.1016/S2213-8587(22)00003-1

[13] Scapaticci S D’Adamo E Mohn A. Non-alcoholic fatty liver disease in obese youth with insulin resistance and type 2 diabetes. Front Endocrinol (Lausanne) 2021;12:639548. 33889132 10.3389/fendo.2021.639548 PMC8056131

[14] Chen C Zhu Z Mao Y. HbA1c may contribute to the development of non-alcoholic fatty liver disease even at normal-range levels. Biosci Rep 2020;40:BSR20193996. 31940026 10.1042/BSR20193996 PMC6997109

[15] Lan Q Zhang Y Lin F. Association between serum aminotransferases and risk of new-onset cardiometabolic disease in a healthy chinese population: a cohort study. Front Public Health 2022;10:902393. 35757633 10.3389/fpubh.2022.902393 PMC9218741

[16] Fujii H Kawada N Japan Study Group of NAFLD (JSG-NAFLD). The role of insulin resistance and diabetes in nonalcoholic fatty liver disease. Int J Mol Sci 2020;21:3863. 32485838 10.3390/ijms21113863 PMC7312931

[17] Sheth H Bagasrawala S Shah M. The HAALT non-invasive scoring system for NAFLD in obesity. Obes Surg 2019;29:2562–2570. 31016455 10.1007/s11695-019-03880-x

[18] Sagara M Iijima T Kase M. Serum levels of soluble dipeptidyl peptidase-4 in type 2 diabetes are associated with severity of liver fibrosis evaluated by transient elastography (FibroScan) and the FAST (FibroScan-AST) score, a novel index of non-alcoholic steatohepatitis with significant fibrosis. J Diabetes Complications 2021;35:107885 33602617 10.1016/j.jdiacomp.2021.107885

[19] Giandalia A Romeo EL Ruffo MC. Clinical correlates of persistently elevated liver enzymes in type 2 diabetic outpatients. Prim Care Diabetes 2017;11:226–232. 28017576 10.1016/j.pcd.2016.12.001

[20] Shimizu M Suzuki K Kato K. Evaluation of the effects of dapagliflozin, a sodium-glucose co-transporter-2 inhibitor, on hepatic steatosis and fibrosis using transient elastography in patients with type 2 diabetes and non-alcoholic fatty liver disease. Diabetes Obes Metab 2019;21:285–292. 30178600 10.1111/dom.13520

[21] Aso Y Kato K Sakurai S. Impact of dapagliflozin, an SGLT2 inhibitor, on serum levels of soluble dipeptidyl peptidase-4 in patients with type 2 diabetes and non-alcoholic fatty liver disease. Int J Clin Pract 2019;73:e13335. 30810254 10.1111/ijcp.13335

[22] Kinoshita T Shimoda M Nakashima K. Comparison of the effects of three kinds of glucose-lowering drugs on non-alcoholic fatty liver disease in patients with type 2 diabetes: a randomized, open-label, three-arm, active control study. J Diabetes Investig 2020;11:1612–1622. 10.1111/jdi.13279 PMC7610105 32329963

[23] Nguyen G Park SY Le CT. Metformin ameliorates activation of hepatic stellate cells and hepatic fibrosis by succinate and GPR91 inhibition. Biochem Biophys Res Commun 2018;495:2649–2656. 29278707 10.1016/j.bbrc.2017.12.143

[24] Lavynenko O Abdul-Ghani M Alatrach M. Combination therapy with pioglitazone/exenatide/metformin reduces the prevalence of hepatic fibrosis and steatosis: The efficacy and durability of initial combination therapy for type 2 diabetes (EDICT). Diabetes ObesMetab 2022;24:899–907. 10.1111/dom.14650 35014145

[25] Mathew G Agha R for the STROCSS Group. STROCSS 2021: Strengthening the Reporting of cohort, cross-sectional and case-control studies in Surgery. Int J Surg 2021;96:106165. 34774726 10.1016/j.ijsu.2021.106165

